# Pararectus approach to the AO B2.2 pelvic fracture: early functional and radiological outcomes

**DOI:** 10.1007/s00590-022-03216-z

**Published:** 2022-02-04

**Authors:** Yi-Hsun Yu, Chang-Heng Liu, Yung-Heng Hsu, Ying-Chao Chou, I-Jung Chen

**Affiliations:** Department of Orthopedic Surgery, Musculoskeletal Research Center, Chang Gung Memorial Hospital, Chang Gung University, Taoyüan, 33302 Taiwan

**Keywords:** Pelvic fracture, Pararectus approach, Quality of reduction, Radiological outcome

## Abstract

**Purpose:**

The pararectus approach is used to treat acetabular fractures; however, it remains unclear whether it can be used to treat pelvic fractures. This study aimed to examine the outcomes of patients with a pelvic ring fracture treated with this approach.

**Methods:**

Seven patients with AO B2.2 pelvic fractures treated with the pararectus approach were included. Patients’ pain was assessed pre- and postoperatively with a numerical rating scale. Radiological evaluations included inlet and outlet ratios and pelvic symmetry. Functional outcomes, including Merle d’Aubigné and Majeed scores, were also recorded for 12 months.

**Results:**

One patient experienced obturator nerve neuropraxia. Pain scores ranged from 2.3–8.0 to 2.0–3.1 points before and after surgery, respectively. Radiological findings revealed satisfactory outcomes. The maximal gap of the affected ilium reduced from 8.6–20.2 to 0–3.4 mm, from 6.8–17.9 to 0–4.4 mm, and from 3.7–20.3 to 0–3.2 mm in the axial, sagittal, and coronal views, respectively. Based on multiple evaluations, functional outcomes were improved for all patients.

**Conclusion:**

The pararectus approach can be used safely and satisfactorily to treat AO B 2.2 pelvic fractures.

## Introduction

The ilioinguinal approach developed by Letournel [1] is a commonly used technique for treating pelvic ring and acetabular fractures. Its advantages include approach through a muscle-sparing plane, excellent exposure of the anterior column of the acetabulum and the inner surface of the innominate bone, and enhancement of functional recovery [2, 3]. However, manipulation, reduction, and fixation procedures through the second window, which are critical to this approach, tend to put the surrounding femoral vessels and nerves at risk [2]. Additionally, the reduction of a fracture through the ilioinguinal approach involves an outside-in mechanism, which results in a high rate of imperfect reduction of the pelvic ring and acetabular fractures [2, 4].

Several alternatives have been developed to overcome the drawbacks of the ilioinguinal approach and improve outcomes [5–7]. Among them, the anterior intrapelvic approach (AIP, previously known as the modified Stoppa approach) has eliminated the challenges associated with the second window manipulations of the ilioinguinal approach by staying within the retroperitoneal space, making this approach popular and efficacious [8, 9].

Meanwhile, Keel et al. [10] developed another approach, known as the pararectus approach. The pararectus approach combines the advantages of the second window of the ilioinguinal approach and the medial view of the AIP approach by involving five windows. Moreover, compared to the AIP approach, in the pararectus approach, fracture reduction and fixation are enhanced using longer screws because the trajectory of the posterior screws is consistent with the surgical incision, directly toward the ischial spine or posterior inferior iliac spine [11]. Studies on the use of the pararectus approach to treat acetabular fractures have shown non-inferior or similar outcomes compared to those achieved with conventional approaches [10, 12, 13].

As the anterior and posterior columns in the acetabular fracture can be appropriately managed by the pararectus approach, this approach may also be used to treat pelvic fractures, in particular, a displaced iliac wing fracture. This study aimed to examine the surgical outcomes of patients with a specific type of pelvic ring fracture (AO B2.2) that was reduced and fixed with a pararectus approach. We hypothesized that this approach would help achieve the anatomical reduction of the pelvic ring fracture and result in satisfactory radiological and functional outcomes.

## Methods

We included patients with an AO B2.2 pelvic fracture who underwent osteosynthesis between January and August 2020. All the patients were managed as per the Advanced Trauma Life Support protocol during investigation in the emergency department and subsequently transferred to the ordinary ward or intensive care unit, as required. The indications for osteosynthesis were as follows: (1) Fragments displaced more than 10 mm in at least one two-dimensional (2D) view of computed tomography (CT) scan; (2) Asymmetric hemipelvis as determined by Lefarine criteria [14] and the inlet/outlet ratio [15]; and (3) Intolerable pain from the fracture. Osteosynthesis for the pelvic fracture was performed immediately after the patient was hemodynamically stabilized. Subsequently, the rehabilitation protocol was individualized according to the patient’s concomitant injuries and fractures.

### Surgical technique

**Fig. 1:**
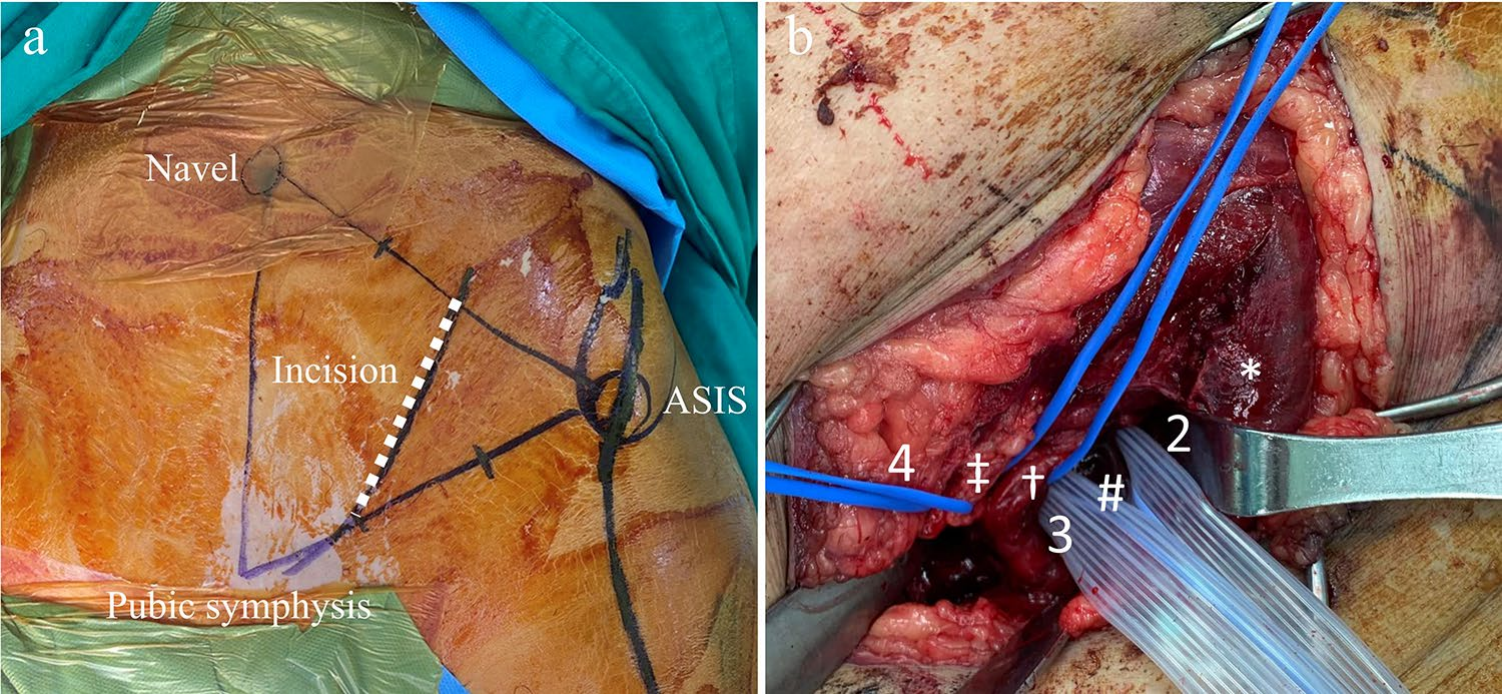
**a** Surgical landmarks for the pararectus approach. **b** The three commonly used windows during surgical dissection. *ASIS* anterior superior iliac spine; *: psoas muscle; #: external iliac vessels; †: spermatic cord; ‡: inferior epigastric artery; 2: second window; 3: third window; 4: fourth window

The pararectus approach used in this study has been previously described [16]. Briefly, the landmarks of the skin incision included the navel, anterior superior iliac spine (ASIS), and pubic symphysis, which formed a triangle. The incision started proximally at the junction of the lateral and middle thirds of the line connecting the navel with the ASIS, continuing as a curve in the distal–medial direction toward the junction of the middle and medial thirds of the line connecting the ASIS with the symphysis (Fig. 1a). Following a superficial incision, layer-by-layer dissection of the Camper’s and Scarpa’s fascia and the external oblique and transversalis muscles was performed to the deepest layers. Five windows were created, as described by Keel et al. [10], as follows: first window: interval lateral to psoas muscle; second window: the interval between the psoas muscle and the external iliac vessel bundle; third window: the superficial interval between the external iliac vessel bundle and vas deference/round ligament and inferior epigastric artery; fourth window: the interval between the inferior epigastric artery and pubic symphysis; fifth window: an interval similar to the 3rd window but deeper into the true pelvis. The first and fifth windows were less explored in this series because the major reduction and manipulation of the AO B2.2 pelvic fracture were achieved within the second, third, and fourth windows (Fig. 1b).

**Fig. 2:**
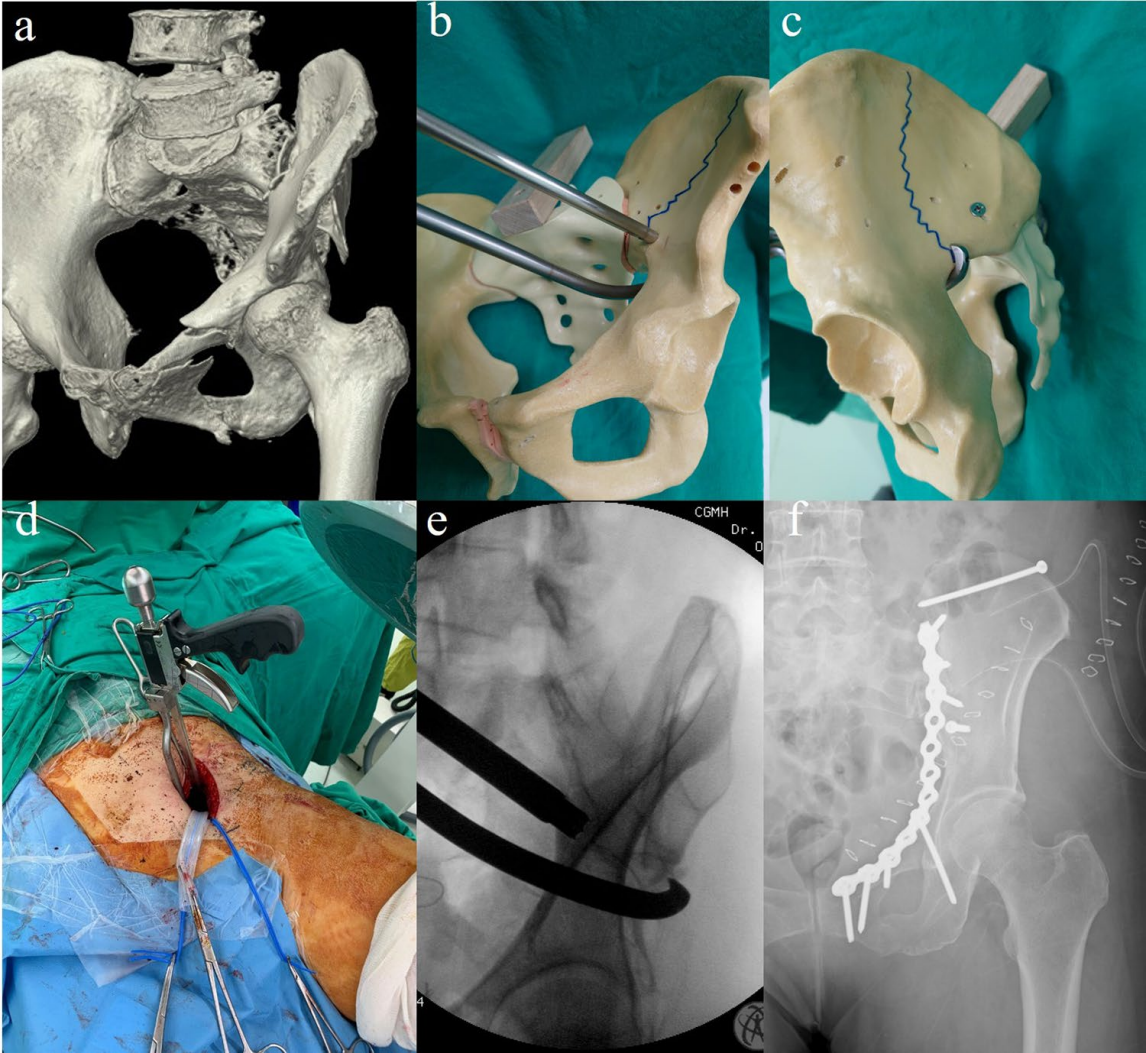
**a** Preoperative obturator oblique view of CT scan. **b**, **c** Illustration of a colinear reduction clamp placement on a pelvic model. **d** Placement of a collinear reduction clamp and **e** the corresponding fluoroscopic image. **f** A lag screw was used to create compression force between the iliac fragments

Once the necessary working windows were prepared, the major reduction procedure was started from the displaced and fractured iliac wing. A collinear reduction clamp (DePuy Synthes, Paoli, PA, USA) was applied through the second window, and the tip of the pelvic arm was hooked to the intact ilium by passing through the greater sciatic notch (Fig. 2a, b). The correct placement of the clamp and precise reduction of the fracture after squeezing the trigger were confirmed by a fluoroscopic examination. A 3.5–mm-lag screw (DePuy Synthes) was usually adequate to hold and compress the fragments (Fig. 2c). A Kirchner wire was used if the fragments were too small for a 3.5 mm screw. The clamp was subsequently released, and the reduction was examined by fluoroscopy.

**Fig. 3:**
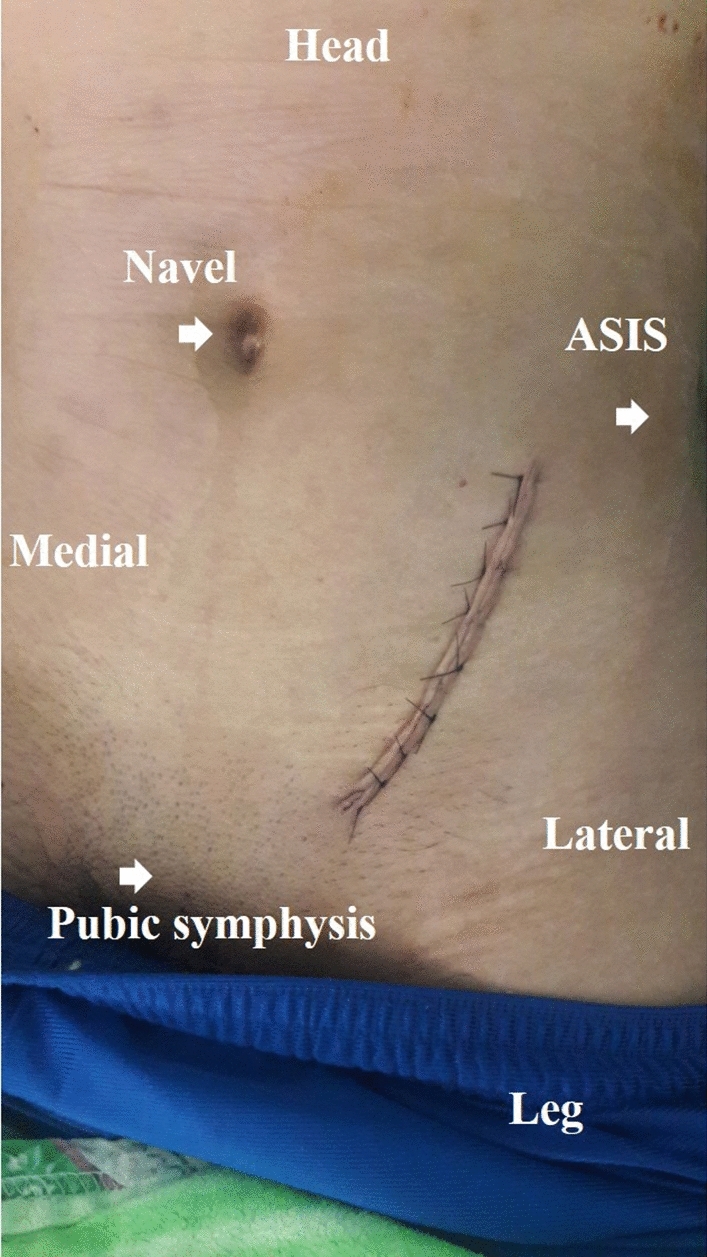
Sutured skin incision following the use of the pararectus approach to treat an AO B2.2 pelvic ring fracture

After the reduction of the iliac crest was completed, the working window was shifted to the fourth window. The presentation of the B2.2 fracture in the superior pubic ramus may be simple, oblique, or segmental. The location of the pubic fracture, which varied among patients, was categorized based on Nakatani classification [17]. The fractured pubic ramus could be reduced by a Weber clamp (DePuy Synthes) in a simple fracture type or bridged with a reconstruction plate (Depuy Synthes) in comminuted fractures. In some cases of a displaced iliac crest, another incision above the iliac crest (first window) was required. The displaced iliac crest could be reduced by a Farabeuf (DePuy Synthes) or Weber clamp and subsequently fixed with an intramedullary lag screw. Once all the fragments were reduced, a 3.5 mm pre-contoured reconstruction plate (DePuy Synthes) was placed along the pelvic brim. Finally, the wound was closed in layers without a drain (Fig. 3). All osteosynthesis and perioperative care were performed by a single surgeon (Y.-H. Yu) during an 8 month period.

### Radiological evaluations

Routine radiological images, including pelvic anteroposterior, inlet, and outlet views, as well as pelvic computed tomography (CT) scans, were obtained to evaluate outcomes. Although several parameters have been proposed for outcome evaluation after osteosynthesis for pelvic fractures [14, 15, 18], this study adapted one of such parameters (Lefaivre’s method), as the fracture of interest is characterized by a major rotational instability. X-ray images were used to assess the symmetry of the pelvis after osteosynthesis using Lefaivre’s criteria. Accordingly, pelvic symmetry was evaluated immediately after osteosynthesis by comparing the distances between the inferior aspects of the sacroiliac joint and the contralateral teardrop on both sides of the pelvis. The difference on both sides of < 5 mm, 5–10 mm, 10–20 mm, and > 20 mm represented excellent, good, fair, and poor symmetry, respectively.

Additionally, we evaluated the inlet and outlet ratios, as proposed by Sagi et al. These ratios were designed to examine the residual translations of the affected hemipelvis. When measuring the inlet ratio, a line perpendicular to the spinal process was drawn across the anterior border of the sacrum on inlet view, and the distance from this line to the subchondral bone of each acetabulum was determined. Afterward, a ratio was calculated for these distances, with the affected extremity being the numerator. A similar method was used to calculate the outlet ratio for the outlet view; however, the baseline was drawn parallel to the superior endplate of *S*1 and perpendicular to the spinous processes. A ratio of 1.00 represents a balance between both sides. A ratio of < 1.00 suggests that the injured hemipelvis is translated posteriorly, superiorly, or in both directions. Meanwhile, a ratio of > 1.00 suggests that the injured hemipelvis is translated anteriorly, inferiorly, or in both directions. The inlet and outlet ratio assessments were performed before and immediately after surgery and then again at 3 months after surgery to assess rehabilitation progress.

Finally, the quality of pelvic fracture reduction was assessed using CT scans in the axial, coronal, and sagittal planes to determine the maximum residual gap. The gaps in all three directions were compared before and immediately after osteosynthesis to determine the improvement in the fracture gap.

To reduce interobserver reliability, all image interpretations were conducted by two surgeons (C.H. Liu and Y.-H. Hsu). If there was a discrepancy between these interpretations, a third surgeon (Y.-H. Yu) would make the final decision.

### Pain evaluation, rehabilitation protocol, and functional evaluations

We used a numerical rating scale (NRS) score to evaluate the pain of the enrolled patients before and after surgery. The preoperative NRS score was the average of the scores recorded by the nursing staff in the period between admission and surgery. Post-surgically, the NRS score was evaluated daily until discharge and presented as the average score.

The postoperative rehabilitation protocol was individualized according to the patients’ demands and concomitant injuries. Usually, a crutch- or walker-assisted toe-touch ambulation was allowed for 4 weeks after osteosynthesis. Following toe-touch ambulation, a progressive increase in weight-bearing during ambulation was introduced. The goal of gait training was to achieve assistance-free ambulation within 12 weeks. Mechanical prophylaxis was applied with compression socks for at least 12 weeks. No routine medical prophylaxis for heterotopic ossification was prescribed.

Functional outcomes were assessed using Merle d’Aubigné [19] and Majeed [20] scores at 3, 6, and 12 months after injury in all patients. The Merle d’Aubigné score includes parameters for pain, mobility, and walking ability, with each parameter rated from 0 points (worst condition) to 6 points (best condition); a high score represents a good hip function. The Majeed score is a pelvic injury-specific functional assessment that comprises seven items, including pain, work, sitting, sexual intercourse, standing, unaided gait, and walking distance, with a total score range of 0–100, in order of decreasing disability.

## Results

Seven patients (one male individual) were included. The patients’ characteristics are presented in Table 1. Five and two patients experienced motorbike accidents and falls, respectively. All the patients underwent osteosynthesis for pelvic fractures between two and four days after sustaining the injury, except for one patient (#6). This patient was a 15-year-old female who underwent osteosynthesis on the day of the injury due to ipsilateral displacement of the femoral neck, which required immediate intervention; both fractures underwent simultaneous osteosynthesis. All fractures were located at the affected ilia and pubic rami (except one). Based on the Nakatani classification for pubic rami fracture, three were located at zone II and three at zone III.

**Table 1:** Demographic characteristics of the seven patients treated for pelvic fractures with the pararectus approach

Patient	Sex	Age, years	Trauma mechanism	Injury severity score, points	Associated injuries	Time to surgery (days)	Surgical time (minutes)	Estimated blood loss (mL)	Incision length (cm)	Surgery- related complications	Numerical rating scale score (mean)	
											Before surgery	After surgery
1	Female	61	Motorbike accident	9	Distal radius fracture, scaphoid fracture	4	120	300	9.5	None	7.0	3.1
2	Male	47	Fall from 6 m height	20	Rib fracture, hemopneumothorax, spleen laceration, scapula fracture	4	223	1100	12.0	Obturator nerve neuropraxia	4.5	3.0
3	Female	23	Motorbike accident	9	None	2	195	500	8.0	None	3.7	2.5
4	Female	67	Motorbike accident	4	None	4	113	350	9.0	None	2.4	2.0
5	Female	19	Motorbike accident	4	None	2	107	150	7.5	None	3.0	2.0
6	Female	15	Fall from 3 m height	9	Ipsilateral femoral neck fracture	0	313	800	7.5	None	8.0	2.0
7	Female	66	Motorbike accident	9	None	3	154	500	8.0	None	2.3	2.0

Overall, the incision length ranged from 7.5 to 12.0 cm. One patient experienced medial thigh pain and sensory loss and weakness with leg adduction after osteosynthesis; hence, postoperative obturator nerve neuropraxia was diagnosed. The symptoms resolved completely within 2 months. NRS scores ranged from 2.3–8.0 to 2.0–3.1 points before and after surgery, respectively. The follow-up period was 12.9 months (range: 8–18 months). The functional follow-ups of the seven patients are shown in Table 2. Although initial functional scores varied among individuals, the subsequent scores revealed their improvement.

**Table 2:** Functional evaluations of the seven patients within 12 months

Patient	Merle d’Aubigné score^a^			Majeed score^b^		
	3 months	6 months	12 months	3 months	6 months	12 months
1	7	12	13	50	59	91
2	14	17	18	64	88	95
3	7	12	15	43	60	77
4	6	18	18	44	88	95
5	3	12	16	44	51	87
6	4	8	12	23	40	66
7	6	15	18	49	62	–

^a^Merle d’Aubigne´ score was graded as excellent (score ≥ 16), good (score of 11–15), fair (score of 6–10), and poor (score ≤ 1–5)

^b^Majeed score was graded as excellent (score > 85), good (score of 70–84), fair (score of 55–69), and poor (score < 55)

Table 3 presents the findings of the pre- and post-surgical X-ray evaluations. Pelvic symmetry was good and excellent in one and six patients, respectively. The preoperative inlet and outlet ratios ranged from 0.89–0.96 to 0.85–1.88, respectively. After osteosynthesis, these ratios improved to 0.98–1.02 (inlet ratio) and 0.96–1.02 (outlet ratio). The inlet and outlet ratios evaluated immediately postoperatively and at 3 months postoperatively were similar.

**Table 3:** Pre- and post-surgical X-ray evaluations of the seven patients treated with the pararectus approach

Patient	X-ray parameter						
	Inlet ratio			Outlet ratio			Reduction quality (Lefaivre’s criteria^a^, mm)
	Before surgery	After surgery	3 months after surgery	Before surgery	After surgery	3 months after surgery	
1	0.90	1.01	1.00	0.96	0.97	0.95	Excellent (4.8)
2	0.96	1.02	1.00	0.94	0.98	0.98	Excellent (3.6)
3	0.91	0.98	0.99	0.95	0.96	0.98	Excellent (4.5)
4	0.92	1.00	0.98	0.85	1.02	1.01	Excellent (1.7)
5	0.89	0.97	0.97	1.88	0.97	0.97	Excellent (1.8)
6	Unavailable^b^	1.01	1.02	Unavailable^b^	0.99	0.97	Excellent (0.1)
7	0.94	1.01	1.01	0.93	1.03	1.03	Good (6.8)

^a^Excellent: < 5 mm, good: 5–10 mm, fair: 10–20 mm, poor: > 20 mm

^b^Surgical intervention for this patient was performed on the day of injury. The inlet and outlet pelvic X-rays were not obtained before osteosynthesis

The postoperative CT findings are summarized in Table 4. The fracture gaps of the ilium ranged from 8.6–20.2 mm, 6.8–17.9 mm, and 3.7–20.3 mm in the axial, sagittal, and coronal views, respectively, at the time of admission. After osteosynthesis, the corresponding values were 0.0–3.4 mm, 0.0–4.4 mm, and 0.0–3.2 mm, respectively. The corresponding rates of gap reduction ranged from 48–83%, 52–100%, and 68–100%, respectively.

**Table 4:** Pre- and post-surgical computed tomography assessments of the seven patients treated with the pararectus approach

Patient	Maximal gap on computed tomography (mm)								
	Axial view			Sagittal view			Coronal view		
	Before surgery	After surgery	Improvement (%)	Before surgery	After surgery	Improvement (%)	Before surgery	After surgery	Improvement (%)
1	10.8	1.8	83	8.2	0.95	88	14.6	0	100
2	20.2	0	100	11.8	0	100	3.7	0.1	97
3	17.9	2.5	86	9.1	4.4	52	20.3	0	100
4	8.6	1.9	72	8.6	1.1	87	8.8	0	100
5	6.6	3.4	48	6.8	2.8	59	7.4	1.9	74
6	19.1	2.4	87	17.9	0	100	14.0	3.2	77
7	10.1	3.2	68	12.2	3.3	73	3.1	1	68

## Discussion

The optimal treatment for a B2.2 pelvic fracture remains unclear. A minimally displaced fracture can be treated either non-surgically or surgically, depending on the case requirements and surgeon’s experience, as both approaches yield similar pain- and ambulation-related outcomes [16, 21]. However, surgical intervention is required for a displaced pelvic fracture; it is associated with pain reduction, pelvic stability and symmetry restoration, and reinstatement of function [22, 23]. Herein, we observed that the average NRS score was improved, stability and symmetry of the pelvis were achieved, and patients could ambulate independently 12 weeks after osteosynthesis. These findings suggest that the pararectus approach may be suitable for treating patients with displaced B2.2 pelvic fractures.

The pararectus approach was originally developed to treat complex acetabular fractures; however, presently, it is also used in other contexts [24–27]. This approach provides better visualization of the false pelvis than that achieved by either the AIP or ilioinguinal approach, extending from the lateral border of the sacrum, sacroiliac joint, ilium, and pubis to the pubic symphysis [11]. Additionally, the fractured ilium can be visualized, and the reduction maneuvre can be applied directly to the fracture. Furthermore, the pararectus approach permits the use of longer posterior columnar and infra-acetabular screws, which in turn, helps secure the stability of osteosynthesis within a small surgical wound [11].

Reducing the fracture gap, restoring the symmetry of the pelvis, and avoiding iatrogenic injuries to the vital organs are critical to osteosynthesis for pelvic fractures. Consequently, one of the aims of the present study was to use surgical instruments to help achieve a reduction in a straightforward and safe manner. By definition, pelvic ring type B2.2 fractures involve a lateral compression force that produces a crescent fracture of the ilium with an internal rotation deformity [28]. The connection between the anterior and posterior iliac segments could be manipulated and reduced using instruments such as a ball-spike pusher, Matta clamps, or a collinear reduction clamp. In our series, after debriding the hematoma between the fractured segments, a collinear reduction clamp was applied perpendicularly to the surgical wound and fracture line without tension to the psoas muscle or abdominal skin, which may occur in the AIP approach. This clamp may also be placed after medial retraction of the external iliac vessels under direct vision, thus preventing iatrogenic injuries to the vital vessels, which may be encountered during manipulation through the second window of the ilioinguinal approach. Furthermore, after reducing the gap with the collinear reduction clamp, a lag screw could be applied easily along with the clamp to create a compressive force between the segments and easily for subsequent plate osteosynthesis.

Although the pararectus approach was reported to have excellent clinical outcomes, some approach-related complications may exist. Specific intraoperative complications related to this approach include injury to epigastric and obturator vessels, peritoneal perforation, and lymphatic leakage during external vessels exploration [4, 10, 12]. With careful surgical dissection for exploration of the five windows, these complications can be avoided. In our limited experience, there have been no such complications during osteosynthesis. However, one patient developed postoperative obturator neuropraxia. The obturator nerve is formed from the lumbar plexus, descends through fibers of the psoas major, and travels toward the obturator foramen of the pelvis. We postulate that the obturator nerve damage might have been due to either placement of the malleable retractor into the obturator foramen within the fourth window or iatrogenic nerve injury from drilling. This study has some limitations that should be considered when interpreting its findings. First, there are several approaches for treating this type of fracture pattern. This study did not compare outcomes associated with these different approaches; however, the most suitable approach is likely the one that is individualized to each patient accounting for surgical history, body weight, and concomitant injuries. This study aimed to explore the advantages of the pararectus approach and provide preliminary evidence on its use in treating a specific fracture pattern; the presented outcomes were satisfactory. Second, some evaluation parameters used in this study were based on X-ray image evaluations. Differences in patient positioning and measurement inconsistencies may have biased the present findings. Nevertheless, Lefaivre’s method and inlet/outlet ratio estimates do not consider the midline; rather, they use the most stable and typically visible landmarks in the pelvic radiograph [26]. Moreover, postoperative CT scans were used to evaluate fracture gap reductions. Therefore, the measurements of the residual fracture gap would be convincible. Finally, long-term follow-ups were lacking. Therefore, future studies should include a greater number of patients and report their long-term functional outcomes. Patients with such a fracture pattern should be followed continuously to further explore the advantages of this approach.

## Conclusion

The indications for the pararectus approach are continuously expanding. This approach may allow unrestricted placement of the reduction clamp, thus helping to achieve a reduction within a small surgical incision for an AO B2.2 pelvic fracture. Nonetheless, future studies should compare the outcomes associated with different treatment approaches for this type of fracture in larger samples.

## Data Availability

All data generated or analyzed during this study are included in this published article. The datasets used and/or analyzed during the current study are available from the corresponding author on reasonable request.
